# 

**Published:** 1916-09-30

**Authors:** 


### CONTENTS.

PAGE

**The Example of the American Hospital Association** ... ... ... ... ... ... 591

**The Final Court of Appeal** ... ... ... ... ... 592

**Hospital and Institutional News** ... ... ... 593

Alexandra Day Distribution—The David Hollin Nursing Home—A Special Medical Exhibition—The Therapeutic Value of Example—A Suggestive Appeal—A Hospital War Savings Association—Naval Hospital Arrangements—Military Beds in Cottage Hospitals—Victimising a Children's Hospital—Surgical Dressings from the U.S.A.—War Charities Registration—An Important Prosecution Under the Public Health Act—A New Hospital for Face Injuries—Skegness Cottage Hospital—Majority Votes on Workmen's Contributions—Retirement of a Veteran Hospital Chairman—The B.M.A. and the Commission of Investigation—Tasmanian Hospital Pension Scheme—When Quacks Fall Out—Lightening the Load—Milk Deliveries—The Red Cross and British Prisoners: The New Committee—Hot Baths for Soldiers—Newspaper Reading in Mental Hospitals—The Value of Tuberculosis Colonies—The Health of Guernsey—This Week's Drug Market.

**Sir George Newman on the School Child** ... 599

The Arousing of the National Conscience.

**A Municipal Milk Supply** ... ... ... ... 600

**Life and Death: Some Human Lessons in War-Time** ... ... ... ... ... ... 601

II.—The Physician and the Preacher.

PAGE

**A Leading Case at Edinburgh** ... ... ... 600

**National Insurance and the Voluntary Hospitals** ... ... ... ... ... ... 603

St. Thomas's Hospital.

**Patriotism in British Hospitals** ... ... ... 604

Guy's Hospital.

**The Matrons' and Sisters' Department** ... 605

Mental Nurses in Military Hospitals.

Round the Hospitals.

The Supply of Nurses Committee: Action by the College of Nursing—Miss Rundle at Plymouth—The Envious Temperament—Scant Courtesy for Ward Sisters—Co-operation in Kingsland Road.

**The Canadian Casualty System** ... ... ... 607

The Work of the Canadian Medical Service.

**Passing Events and Topics** ... ... ... ... 608

**Hospital and Institutional Results in 1915** ... 608

**Oliver Wendell Holmes** ... ... ... ... 609

A Medical Writer Who Missed his Vocation.

**Gifts to Hospitals** ... ... ... ... ... 610

**Matrons' and Sisters' Appointments** ... ... iv

**The Book Market** ... ... ... ... ... iv

**Gallantry of R.A.M.C. Officers** ... ... ... iv

**The Institutional Worker** ... ... (Suppl.) 1

Saving the Babies.

